# Self-evaluation as an active ingredient in the experience and treatment of adolescent depression; an integrated scoping review with expert advisory input

**DOI:** 10.1186/s12888-021-03585-5

**Published:** 2021-12-03

**Authors:** Faith Orchard, Juliette Westbrook, Brioney Gee, Tim Clarke, Sophie Allan, Laura Pass

**Affiliations:** 1 School of Psychology and Clinical Language Sciences, University of Reading, Reading, UK; 2 School of Psychology, University of Sussex, Brighton, UK; 3 Research and Development Department, Norfolk and Suffolk NHS Foundation Trust, Norwich, UK; 4 Department of Clinical Psychology and Psychological Therapies, Norwich Medical School, University of East Anglia, Norwich, UK

**Keywords:** Depression, Adolescence, Self, Youth involvement

## Abstract

**Background:**

Negative self-perceptions is one of the most common symptoms of depression in young people, and has been found to be strongly associated with severity of depression symptoms. Psychological treatments for adolescent depression are only moderately effective. Understanding the role and importance of these self-perceptions may help to inform and improve treatments. The aim of this review was to examine self-evaluation as a characteristic of adolescent depression, and as an active ingredient in treatment for adolescent depression.

**Methods:**

We conducted a scoping review which included quantitative and qualitative studies of any design that reported on self-evaluation as a characteristic of, or focus of treatment for, adolescent depression. Participants were required to be 11–24 years and experiencing elevated symptoms of depression or a diagnosis. We also met with 14 expert advisory groups of young people with lived experience, clinicians, and researchers, for their input. Findings from 46 peer-reviewed research studies are presented alongside views of 64 expert advisors, to identify what is known and what is missing in the literature.

**Results:**

Three overarching topics were identified following the review and reflections from advisors: 1) What does it look like? 2) Where does it come from? and 3) How can we change it? The literature identified that young people view themselves more negatively and less positively when depressed, however expert advisors explained that view of self is complex and varies for each individual. Literature identified preliminary evidence of a bidirectional relationship between self-evaluation and depression, however, advisors raised questions regarding the influences and mechanisms involved, such as being influenced by the social environment, and by the cognitive capacity of the individual. Finally, there was a consensus from the literature and expert advisors that self-evaluation can improve across treatment. However, research literature was limited, with only 11 identified studies covering a diverse range of interventions and self-evaluation measures. Various barriers and facilitators to working on self-evaluation in treatment were highlighted by advisors, as well as suggestions for treatment approaches.

**Conclusions:**

Findings indicate the importance of self-evaluation in adolescent depression, but highlight the need for more research on which treatments and treatment components are most effective in changing self-evaluation.

**Supplementary Information:**

The online version contains supplementary material available at 10.1186/s12888-021-03585-5.

## Background

Depression markedly increases in adolescence [1], with 2.6% of young people experiencing depression at any one time [2], with a cumulative frequency of depression over the course of adolescence rising to as high as 20% by the age of 18 years [3]. The experience of depression in youth can have significant long-term implications [4], including further episodes of depression as an adult [5, 6], the development of other mental health disorders [7, 8] and an increased risk of suicidal behaviour [9]. Adolescent depression has also been associated with functional impairment across the life course such as educational underachievement, low income levels and unemployment [10].

Negative self-perceptions, including worthlessness, are one of the most common symptoms of depression in young people [11], as well as being a core component of the cognitive model of depression [12]. Beck proposed that depressed individuals view themselves, the world and the future in a negative way and that this ‘cognitive triad’ affects how they think, feel, and act, and consequently maintains their depression. In support of the cognitive model, results have shown that depressed adolescents characteristically use more negative and fewer positive words to describe themselves compared to healthy young people [13–15]. Furthermore, negative self-evaluation severity correlates with depression severity in community samples [16] and can identify depression diagnoses as accurately as a full depression symptom questionnaire [17].

Self-concept develops across the lifespan. During early childhood, children typically have inflated global self-esteem, with unrealistically positive domain specific self-concepts [18, 19]. As cognitive ability advances, global self-esteem decreases, as specific self-concepts are formed with influences from external feedback and social interactions [19, 20]. During adolescence, the evaluation of the ‘self’ becomes increasingly more complex. The ability to engage in abstract thinking allows for a more sophisticated sense of self to develop, incorporating social comparisons and different social roles [20, 21]. Most current research in the context of ‘self’ and adolescent depression has focused on global self-esteem, and little is known about the more specific and complex self-concepts, as well as the role of this developmental pathway to self-concept. Furthermore, even though this work is focused on the ‘self’, the literature drawing on young people’s voices, such as qualitative methods, is extremely lacking, although the studies that have been conducted do suggest that young people place significant importance on the subject even when it is not a direct target of the research [22, 23].

One key challenge with the ‘self’ literature is the diverse range of terminology. Three key terms commonly used are: *self-efficacy* – “a person’s judgments of their capabilities to organize and execute courses of action required to attain designated types of performances” [24]; *self-esteem* – an individual’s confidence in their worth or abilities [25]; and *self-concept* – a view constructed of one’s self, which is developed through experiences and evaluations adopted from others [25]. Many other terms are also used interchangeably. The present study reports on ‘*self-evaluation*’ with our working definition of ‘the perceptions and beliefs that a person holds about themselves, specifically the emotionally-valenced qualities, characteristics and traits (both positive and negative), and the person’s judgement of the value of these attributes’. This overlaps with ‘self-concept’ but is distinct from self-esteem (which refers to a more global view), and self-efficacy (which relates to the ability to produce certain actions or skills).

Current adolescent depression therapies are only moderately effective [26, 27], so understanding the role of self-evaluations may help to inform and improve treatments. This project was carried out as part of the Wellcome Trust Mental Health Priority Area, specifically their review of the “active ingredients” involved in treatment of anxiety and depression in young people. The “active ingredients” terminology draws on a cooking analogy of the key ingredients in a recipe, i.e. those needed for success, considering the individual receiving the treatment, the quantity and quality of the ingredients, and the cost and accessibility of these ingredients. Whilst there was emerging evidence of a key role for self-evaluation in depression from the quantitative literature, to the authors’ knowledge, there was no existing review of self-evaluation in adolescent depression, and the work that has been conducted has been hard to pull together due to the heterogenous terminology used in this field. Furthermore, given the growing recognition of an important role for lived experience input in research, it was decided that a scoping review, supported by consultation with expert advisors regarding the gaps in the literature, would provide the most comprehensive next step for advancing the knowledge regarding whether self-evaluation might be a key ‘active ingredient’ in the treatment of adolescent depression.

A scoping review, according to the PRISMA Guidelines - Extension for Scoping Reviews [28], follow a systematic approach to map evidence and identify main concepts. Scoping reviews can meet various objectives, but in the current case, a scoping review was deemed most appropriate as the authors wanted to establish the size, range and nature of evidence on self-evaluation in adolescent depression, and because the methodology of existing research is heterogenous. This scoping review can also establish the value of undertaking a future full systematic review.

We aimed to conduct a review to examine what is known about self-evaluation as 1) a characteristic of adolescent depression, and 2) an active ingredient in treatment for adolescent depression. This research takes a novel approach by integrating the scoping review with expert advisory input from young people with lived experience, clinicians, and researchers. The review reports on the views of the advisors and how their insights align with, or differ from, the existing literature. On this basis, the following research questions were formulated:

1. What does existing research tell us about self-evaluation as a characteristic of adolescent depression, and as an active ingredient in treatment for adolescent depression?
2. To what extent does the existing research reflect the lived experience of self-evaluation and depression, according to experts by experience?

## Methods

### Systematic literature search

The review was conducted in accordance with PRISMA guidelines for scoping reviews [28], and the 22-item checklist has been included as supplementary material. A PROSPERO registration form was utilised to help establish methodology although it was not able to be published on the website due to the scoping nature of the review. The protocol can also be found in supplementary material.

#### Search strategy

We searched five electronic databases (WebofScience, EMBASE, PsychINFO, Medline, The Cochrane Library) from inception to 17th July 2020. The search string was developed based on a preliminary search of WebofScience and scoping searches, and adapted based on suggestions from advisor events.

The following search terms were used: (self-evaluat* OR self-concept OR self-worth OR self-aware* OR self-inhibiting OR “view of self” OR self-assessment* OR “positive evaluation” OR “negative evaluation” OR “positive self” OR “negative self” OR self-reflect* OR self-description OR cognitive-evaluation OR “self-referential processing” OR self-criticism OR self-perception OR self-cognition OR “cognitions about the self” OR self-schema* OR self-image OR “sense of self” OR self-identity OR self-representation OR self-belief* OR self-efficacy OR self-hat* OR self-appraisal) AND (depress* OR MDD OR “low mood”) AND (adolescen* OR teen* OR youth* OR young OR student* OR child* OR pupil* OR juvenile* OR “emerging adult”).

#### Eligibility criteria

Both quantitative and qualitative studies of any design that reported on self-evaluation as a characteristic, or focus of treatment, were eligible. Participants were required to be 11–24 years old to reflect current views of adolescent and ‘young people’ age range [29], experiencing elevated symptoms of depression or a depression diagnosis. Full inclusion and exclusion criteria are outlined in Table 1.

**Table 1:** Inclusion and exclusion criteria

	Inclusion	Exclusion
**Participants**	All participants must be between the ages of 11 to 24. If only mean and SD is given, mean+/− SD must fall within our target age range. If age is not specified, include ‘adolescent’	If age is not mentioned, exclude ‘adults’, ‘children’, ‘infants’, ‘students’ Specific participant group that may present with unique self-evaluation e.g. all homeless
**Depression status**	Primary diagnosis of depression. Identified through prior diagnosis, clinical interview, or meet threshold for elevated depression symptoms prespecified by the study authors	Median split depression measures. Thresholds identified not relating to clinical cut offs, e.g. no justification for cut off.
**Co-occurring conditions**	Where studies report on a participant group with primary anxiety or bipolar II, and secondary depression.	Where other physical or mental health conditions are reported as the primary problem.
**Self-evaluation measurement**	Any study that measures self-evaluation (or related terms) as a characteristic, or target of intervention, in adolescent depression.	Where the only measurement of self is: 1) too broad e.g. self-esteem; 2) too specific e.g. self-efficacy that focuses on the evaluation of a specific skill 3) not relevant to the self-judgement of the individual e.g. self-awareness 4) a more generic measure with self-items but no subscale e.g. a depression measure
**Type of study**	Peer-reviewed primary research.	Abstract, protocol, grey literature, systematic reviews, meta analyses
**Language**	English only	All other languages

#### Study identification and data charting

The first 5% of titles and abstracts were double screened by FO and JW, and discrepancies resolved through discussion with a third independent reviewer (BG). The inclusion criteria were further operationalised, then titles and abstracts screened by one of the team members. Full text reviews were double rated by FO and JW independently, discrepancies discussed and resolved with two independent reviewers (BG, LP). Finally, data was double-extracted, independently by two reviewers (FO, JW), and cross-referenced for discrepancies. Forms were drawn up for data extraction, identifying: author, year, number of participants, % female, age, population, study design, intervention type, measure of depression, type of self that is examined e.g. self-concept/self-evaluation, the measure or tool used to examine self, and key findings. Extracted data was then checked for accuracy and clarity by a third reviewer (LP).

### Expert advisory groups

To address research question two, expert advisors were sought, including young people with experience of low mood or depression, clinicians with experience of working with adolescents with depression, and researchers with relevant expertise. The purpose of the expert advisors was to inform whether the identified research reflected the experience of relevant ‘experts’ and to help identify any gaps in the literature. These consultations did not constitute primary research and as such ethical approval was not obtained to conduct this exploration.

#### Advisory group attendees

Expert advisory groups of young people with lived experience of depression (*n* = 25), clinicians working with adolescents with depression (*n* = 30), and child/adolescent mental health researchers with relevant expertise (*n* = 9), were recruited. Young people with lived experience were contacted via existing, established lived experience advisory groups in the UK. Communication was initiated via group leads, who shared the opportunity with their networks. Groups were informed that we were looking for young people aged 11–24, with experience of low mood or depression. Clinicians and researchers were contacted via existing networks, personal communications and social media. Table 2 outlines the number of events and attendees.

**Table 2:** Expert Advisory Groups Overview

	Number of Events	Number of Advisors	Advisory group details	Facilitators
**Researcher**	2	9	*n* = 8 (1 psychologist, 6 clinical psychologists, 1 psychiatrist; 6 from UK, 2 from Australia)	FO, LP, BG, JW
			*n* = 1 (clinical psychologist; from UK)	FO
**Clinician**	5	30	*n* = 7 (psychological therapist; assistant practitioner; counsellor; cognitive-behavioural therapist; interpersonal psychotherapist; eye movement desensitisation and reprocessing therapist; psychiatrist; clinical psychologist)	TC, LP, JW
			*n =* 6 (commissioning group clinical lead; Social worker CAMHS practitioner; consultant psychiatrist; school nurse; CAMHS psychiatrist; CAMHS inpatient/home treatment team psychiatrist)	TC, LP, JW
			*n =* 5 (counselling psychologist; counsellor; CAMHS clinical lead; mental health support team in schools supervisor; IPT-A therapist)	TC, LP, JW
			*n =* 7 (occupational therapist; mental health nurse; children’s wellbeing practitioner; CBT therapist; senior supervisor clinician; clinical psychologist; IPT therapist)	TC, LP, JW
			*n = 5* (CBT therapist and senior mental health nurse; primary mental health worker; CBT therapist and lead; GP; mental health nurse)	TC, JW
**Young person**	7	25	*n = 5*	BG, SA, JW
			*n =* 10	BG, SA, JW
			*n = 1*	SA
			*n* = 1	BG, JW
			*n = 6*	BG, SA, JW
			*n = 1*	SA
			*n* = 1	SA

#### Procedure

**Fig. 1:**
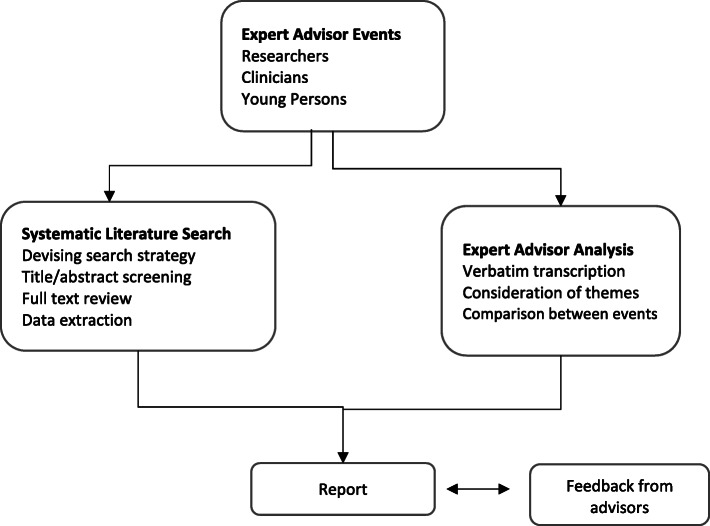
Overview of Procedure

Figure 1 outlines the procedure overview. Expert advisory meetings took place using virtual video conferencing, facilitated by members of the research team.

At the start of each meeting, the purpose of the project, and an explanation of the active ingredient, was provided. Given the complex nature of self-evaluation, and the lack of research specifically on self-evaluation as an active ingredient in improving depression outcomes, four main topics were discussed to help elicit expert views: 1) What is your understanding of self-evaluation? 2) How do young people describe / talk about themselves as part of assessment and treatment for adolescent depression? 3) Is self-evaluation currently targeted as part of treatment, and does self-evaluation change throughout treatment, even if not targeted? And 4) Should interventions more directly target self-evaluation, and if so, how should this be done?

After advisory events were completed and the systematic literature review was complete, integrated findings were shared with expert advisors and minor revisions made.

#### Data extraction and analysis

Advisory groups were audio recorded, transcribed verbatim, read and re-read to ensure familiarity. Transcripts were reviewed by FO and JW, then interpretations and themes were compared and discussed. Themes were then taken to the remaining event facilitators for further discussion and consensus.

### Strategy for synthesis of literature and expert advisor reflections

Scoping reviews aim to map key concepts, types of evidence and gaps in research related to a defined research topic by systematically searching, selecting and synthesising existing literature. To achieve these aims, our approach to data synthesis combines a descriptive quantitative summary of the extent of the literature with a narrative description of study findings. In line with published recommendations for scoping reviews [30, 31] and the requirements of the funder, the insights of stakeholders, including young people with lived-experience of depression, will be integrated with the narrative summary.

## Results

**Fig. 2:**
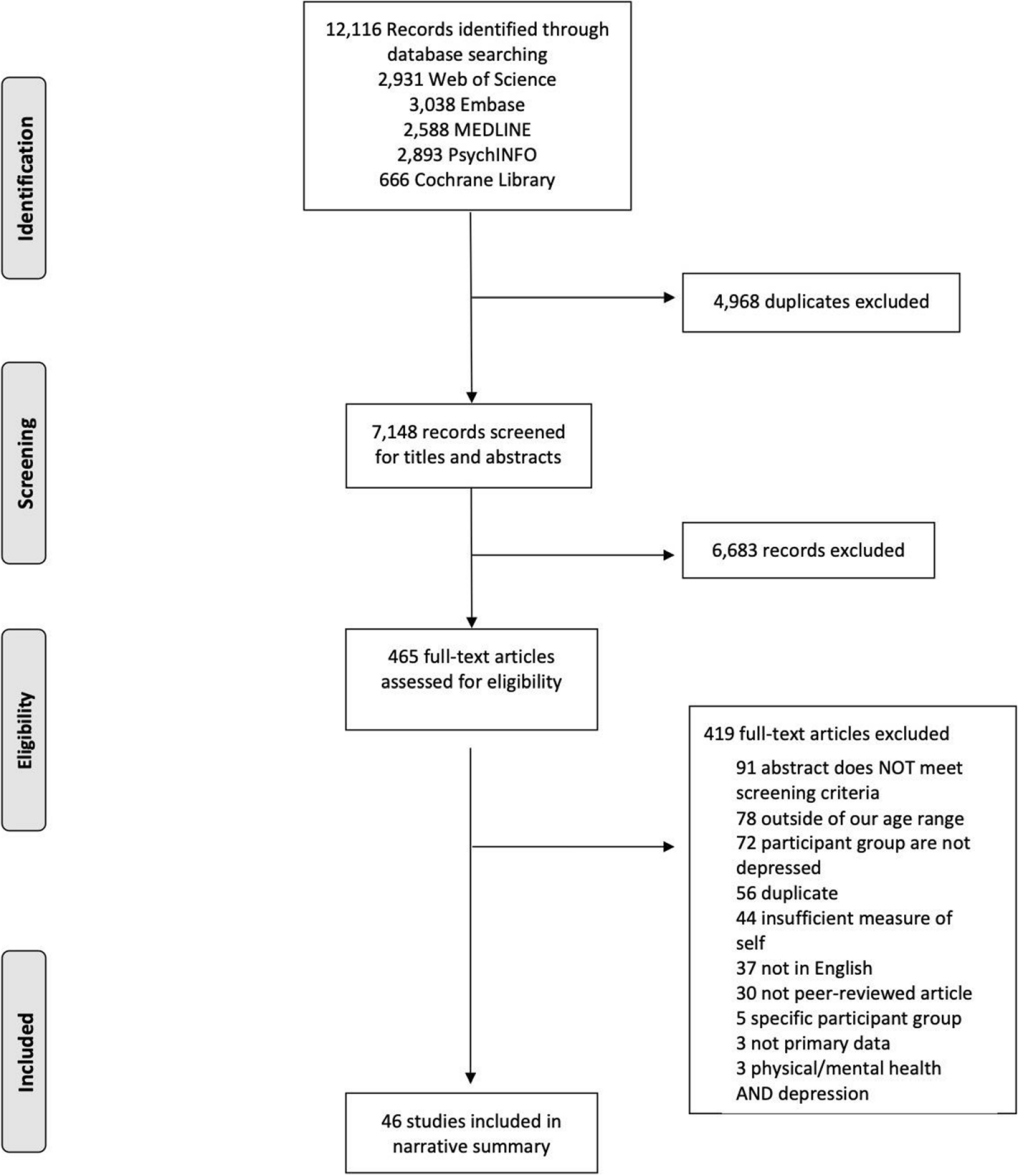
PRISMA Flow Diagram

The study selection process is illustrated in Fig. 2. We identified 46 unique studies that met the inclusion criteria; characteristics and results of included studies are presented in Table 3.

**Table 3:** Characteristics of included studies

**Cross-sectional studies**									
**Study**	**N (depressed)**	**% Female**	**Age in years**	**Recruitment**	**Self Terminology**	**Self Measure**	**Depression Status**	**Effects**	
Auerbach et al. (2015) [13]	52 (22)	100	13–18	Community	Self-referential biases and self-criticism	CTIC-S & endorsement of positive and negative words	Diag. (KSADS; DSM-IV)	Depression associated with more positive and fewer negative words.	
Becker-Weidman et al. (2009) [32]	439	54	12–17	TADS trial participants	View of self	CTIC-S	Diag. & Elev. (DSM-IV and CDRS-R ≥ 45)	Depression and hopelessness associated with view of self.	
Bennett et al. (1997) [33]	328	58	11–19	Outpatient and inpatient	Self-attitude	BDI (negative self-attitude factor)	Diag. (KSADS; DSM-III-R)	Depression associated with worse negative self-attitude than anxiety, disruptive behaviour and other psychiatric controls.	
Bradley et al. (2016) [34]	41 (23)	56	12–20	Outpatient and community	Self-perception	Self-referent judgement	Diag. (KSADS; DSM-IV)	Depression associated with more positive and fewer negative descriptions.	
Cooper et al. (2005) [35]	272 (28)	100	17–18	Community	Core beliefs	YSQ and negative self-beliefssubscale of the EDBQ	Elev. (Median split BDI)	Depression associated with more negative self-beliefs than eating disorder and healthy control group. Endorsement of negative word types varied between groups.	
Dozois et al. (2012) [36]	47 (22)	60	13–17	CAMH program and community	Core beliefs and self-concept	YSQ–Short Form & HSPCA	Diag. (DICA-IV; DSM-IV)	Mixed effects. Depression associated with some self-concepts compared to non-psychiatric controls.	
Grilo et al. (1999) [37]	127 (53)	70	12–18	Inpatient	Self-criticism	DEQ-A	Diag. & Elev. (DSM-III-R and BDI ≥18)	Mixed effects. Depression associated with higher self-criticism than abused group, but no difference on dependency subscale.	
Heath et al. (1999) [38]	104 (29)	47	M = 170.9 Months (SD = 8.74)	School students	Self-concept	SPPC	Elev. (CDI ≥12)	Mixed effects. Depression associated with worse academic and non-academic self-concept. Age differences identified.	
Kendall et al. (1990) [39]	34	55	11–13	School students	Self-evaluation	My Standards Questionnaire	Diag. (KSADS; DSM-III)	Depression associated with lower evaluation of performance on personal domains.	
Koenig (1988) [40]	721 (213)	50	12–19	School students and inpatients	Self-image	OSIQ	Diag. (Patient medical record; DSM-III)	Recurrent depression associated with poorer self-image than dysthymic disorder or atypical depression, but better self-image than single episode of depression for younger participants aged 12–15	
Korhonen et al. (2001) [41]	107 (68)	73	M = 17.9 (SD = 2.3)	Outpatient facility	Self-image	OSIQ	Diag. (SCID; DSM-III-R)	Mixed effects. Depression associated with worse self-image according total scores. Majority of subscales worse in depressed group.	
Lopez Molina et al. (2014) [42]	137	74	18–24	Community	Self-criticism	BDI items	Diag. (MINI; DSM-IV)	Mixed effects. Depression in females associated with some higher scores of self-criticism than depression in males.	
Marton et al. (1993)	103 (38)	52	15–19	Outpatient facility and schools	Self-Perception	HSPPA	Diag. (KSADS; DSM-III-R)	Mixed effects. Depression associated with some lower scores of self-perception compared to control clinical and healthy groups.	
McClure et al. (1997) [43]	31 (14)	100	12–17	School students	Self-Perception	HSPPA	Diag. (DICA-R-A)	Mixed effects. Depressed group rated themselves as less competent on some subscales of self-perception.	
Morey-Nase et al. (2019) [22]	11	64	17–24	Outpatient facility	Self-esteem	Qualitative interview	Diag. (Clinician diagnosis)	Young people described feeling like they are letting people down, not meeting own expectations, disappointing others, and self-loathing.	
Ofonedu et al. (2013) [44]	10	60	13–17	African American School students	Self	Qualitative interview	Diag. (K-SADS; DSM-IV)	Themes emotional sense of self, survival self and healing self. This included experiences of feeling worthless, inadequate, stupid, ugly.	
Orchard et al. (2017) [11]	100 (43)	85	12–17	NHS CAMHS	Self-perception	K-SADS	Diag. (KSADS)	Depression associated with higher negative self-perception than other clinical group and no diagnosis group.	
Orchard et al. (2019) [45]	291 (33)		12–18	Outpatient facility and schools	Self-evaluation	Self-description questionnaire	Diag. (KSADS; DSM-IV)	Depression associated with more negative and fewer positive words. Factor analysis revealed pro-social words which were equally endorsed by depressed and community adolescents.	
Pilecki et al. (2008) [46]	90 (36)	100	Adolescent girls	Outpatient unit and school students	Self-image	OSIQ	Diag. (DSM-IV)	Mixed effects. Depression associated with worse self-images on majority of subscales, and worse self-image than anorexia on some subscales.	
Pinto et al. (1996) [47]	40 (21)	100	13–17	Adolescent inpatient unit	Self-concept	PHCSCS	Diag. (DICA-R-A; DSM-III-R)	Mixed effects. Depression associated some negative self-concepts. BPD with depression group reported lower self-concept on some scales compared to depressed non-BPD group.	
Qian et al. (2002) [48]	79 (40)	56	M = 20	Undergraduate	Self-evaluation	SAI	Elev. (BDI ≥ 13)	Depression associated with lower self evaluation and lower perceived efficacy.	
Quevedo et al. (2017) [49]	121 (86)	50	M = 14.75 (SD =1.64)	Inpatient unit and community	Self-attributions	Self-appraisal task	Diag. (KSADS; DSM-IV)	Depression associated with more negative and fewer positive self-evaluations. No difference between depressed groups.	
Robinson et al. (1992) [50]	50	56	11–17	Inpatient	Self-concept	PHCSCS	Diag. (Hospital records)	Mixed effects. Depression associated with some subscales of self-concept.	
Ross (1989) [51]	33 (18)	51	18–22	University students	Self-traits	Self-referent judgement	Elev. (BDI ≥10)	Depression associated with more unstable positive and negative endorsements, i.e. endorsement of traits was more likely to change.	
Ross et al. (1986) [52]	72	–	18–22	University students	Self-traits	Self-referent judgement	Elev. (BDI ≥14)	Mixed effects. Depression associated with more negative traits, but not less positive traits.	
Rotundo et al. (1985) [53]	84 (22)		12–16	Inpatient, outpatient and schools	Self-esteem and self-perception	PHCSCS	Diag. (DSM-III)	Depression associated with worse self-concept compared to clinical controls.	
Savilahti et al. (2018) [54]	409 (206)	71	13–17	Inpatient and community	Self-image	OSIQ	Diag. (DSM-IV)	Depression associated with worse self-image.	
Wixom et al. (1993) [55]	52 (17)	100	14–18	Inpatient	Self-criticism	DEQ	Diag. (medical chart; DSM-III)	BPD associated with more self-criticism than depression.	
Woo et al. (2004) [56]	480 (238)	40	13–19	Outpatient facility and schools	Self-evaluation	Asian Adolescent Depression Scale	Diag. (KSADS; DSM-IV)	Depression associated with greater negative self-evaluation compared to community and clinical controls	
**Longitudinal studies**									
**Lead Author**	**N (depressed)**	**% Female**	**Age in years**	**Sample**	**Self Terminology**	**Self Measure**	**Depression Status**	**Findings**	
Ames et al. (2018) [57]	662	52	12–18, followed up at 22–29 years	Community based prospective	Physical self-concept	Items from ‘HealthBehaviour in School-Aged Children scale’	Elev. (B-CFPI; ‘Persistent high’ class based on latent class growth analysis’)	Mixed effects. Self-concept differed between different classes at time 1 and over time.	
Carbonell et al. (1998) [58]	108	–	Data at 5,9,15 and 18 years	Community	Self-concept	PHCSCS	Diag. (DSM-III-R)	Mixed effects. Some self-perceptions at age 9 associated with some impaired behavioural academic and psychosocial functioning at age 15.	
Ferro et al. (2015) [59]	2825	49	Data at multiple time points from 10 to 25 years	National Longitudinal Survey of Children and Youth	Self-concept	General Self-Image subscale of the Self-Description Questionnaire (Marsh 1992)	Elev. (CES-D)	Young people on a trajectory of sub-clinical and clinical symptoms showed poorer self-concept over time compared to those with minimal symptoms.	
Fine et al. (1993) [60]	47	85	M = 15.2 (SD = 1.1)	Outpatient facility	Self-concept	OSIQ	Diag. (K-SADS; DSM-III-R)	Self image predicted depressive symptoms/recovery from depression at 3 months and a year. Self-image was better predictor of depression than depression was of self image.	
Franko et al. (2005) [61]	1727 (246 moderate, 209 mild)	100	16–18	Longitudinal cohort study	Self-worth	HSPPA	Elev. (CES-D - 16-23 mild depression, ≥24 moderate depression)	Mild and moderate depressed groups had lower self-worth than the non-depressed group 3 years later.	
**Cross-sectional and intervention**									
**Study**	**N (depressed)**	**% Female**	**Age**	**Sample**	**Self Terminology**	**Self Measure**	**Depression Status**	**Intervention**	**Effects**
King et al. (1993) [62]	60 (30)	76	Inpatient M = 15.8 (SD = 1.1) Outpatient M = 15.7 (SD = 1.2)	Inpatient and community	Self-worth	HSPPA	Diag. (KSADS; DSM-III-R)	multidisciplinary programme	Mixed effects. Depression associated with global self-worth and some subscales. Improvement in depression was marked by increases in global self-worth and some subscales.
**Intervention studies**									
**Study**	**N (depressed)**	**% Female**	**Age**	**Sample**	**Self Terminology**	**Self Measure**	**Depression Status**	**Intervention**	**Effects**
Alavi et al. (2018) [63]	15	86	14–17	Outpatient facility	Self-concept	BSCI-Y	Diag. (DSM-IV)	Face to face vs e-CBT	Mixed effects. e-CBT and F2F did not differ on post-treatment self-concept. Pre- to post-treatment scores did not change in F2F group but did improve in e-CBT.
Fine et al. (1991) [64]	66	83	13–17	Inpatient	Self-concept	OSIQ	Diag. (KSADS; DSM-III-R)	Social skills training vs therapeutic group support	Mixed effects. Improvements in self-concept at post-treatment for therapeutic support group, no change for social skills group. At 9-month follow up social skills group improved.
Gottlieb et al. (2016) [65]	439	54	12–17	TADS treatment trial	Self-concept	CTIC-S	Diag. & Elev. (MDD & CDRS-R ≥ 45	Fluoxetine vs CBT vs combination vs placebo	Mixed effects. Over 12 weeks, combined treatment group outperformed other groups on self-concept. Over 36 weeks combined group only outperformed the fluoxetine group.
Hintikka et al. (2003) [66]	39	61	13–17	Inpatient	Self-image	OSIQ	Diag. (SCID; DSM-III)	Individualised inpatient treatment programmes	Mixed effects. Improvements after treatment on some aspects of self-image.
Kurdziel et al. (2018) [67]	1	100	14	University clinic	Self-esteem and self-criticism	N/A	Diag. (DSM-IV)	Psychodynamic psychotherapy	Long-term psychodynamic therapy discussed as a method for targeting self-criticism amongst problems.
Le Noury et al. (2015) [68]	275	37	12–18		Self-Perception	HSPPA	Diag. (KSADS; DSM-III-R)	Paroxetine (20–40 mg), imipramine (200–300 mg), or placebo.	No effect. Paroxetine and imipramine did not improve self-perception compared to placebo.
Lusk et al. (2011) [69]	15	60	12–17	Outpatient facility	Self-concept	BSCI-Y	Diag. (DSM-IV-TR)	Cognitive–behavioural skills building intervention	Self-concept improved
Nasstasia et al. (2019) [70]	68	53	M = 20.75 (SD = 2.59)	Community and university populations	–	BDI (sub scales and items)	Diag. (SCID-1; DSM-IV)	Initial session of motivational interviewing followed by 12-week, multi-modal exercise program	Mixed effects. Improvement after intervention on some items from the cognitive subscale.
Rickhi et al. (2015) [71]	62	70	12–24 (split into young 12–18 and older 19–24)	Community	Self-concept	Piers Harris II (younger) and (older) SFSCS	Diag. & Elev. (DSM-IV-TR & CDRS-R 40–70 or HAMD 12–24)	Spirituality informed e-mental health tool	Mixed effects, age differences. Self-concept improved for younger participants immediately after the intervention compared to waitlist, and over time. In older participants, change only in one of six factors.
Riley et al. (2011) [72]	7	28	12–16	Inpatient and Outpatient	Self-concept	TSCS-II short form	Diag. (Clinician assigned)	Group therapy. Based on adventure and problem-solving.	Mixed effects. Four out of six participants that completed treatment showed improvement in self-concept. More change in self-concept was seen towards the end of treatment.
Rossello et al. (2008) [73]	112	55	12–18	School students	Self-concept	PHCSCS	Diag. & elev. (DISC-2.1; DSM-III-R or CDI > 13)	Individual/group CBT or IPT	Mixed effects. Self-concept improved in both group and individual CBT but not in the IPT conditions.

NB. Abbreviation key: *DSM* Diagnostic Statistical Manual, *M* Mean, *BPD* Borderline Personality Disorder, *SD* Standard Deviation.

Depression measures: *B-CFPI* Brief Child and Family Phone Interview, *BDI* Beck Depression Inventory, *CDI* Children’s Depression Inventory, *CDRSR* Children’s Depression Rating Scale – Revised, *CES-D* Centre for Epidemiological Studies – Depression Scale, *DEQ* Depressive Experiences Questionnaire, *DEQ-A* Depressive Experiences Questionnaire for Adolescents, *DICA-R-A* Revised Diagnostic Interview for Children and Adolescents, *DISC* Depression Intensity Scale Circles, *HAM-D* Hamilton Depression Rating Scale, *K-SADS* Kiddie-Schedule for Affective Disorders and Schizophrenia, *MINI* Mini International Neuropsychiatric Interview, *MFQ* Mood and Feelings Questionnaire, *SCID* Structured Clinical Interview for DSM.

Self measures: *BSCI-Y* Beck Self-Concept Inventory for Youth, *CTIC-S* Cognitive Triad Inventory for Children View of Self Subscale, *EDBQ* Eating Disorder Belief Questionnaire, *HSPPA/HSPSA* Harter Self-Perception Profile/Scale for Adolescents, *OSIQ* Offer Self Image Questionnaire, *PHCSCS* Piers-Harris Children’s Self-Concept Scale, *SAI* Self-Appraisal Inventory, *SFSCS* Six-factor Rating Scale, *SPPC* Self-Perception Profile for Children, *TSCS-II* Tennessee Self Concept Scale, *YSQ* Young Schema Questionnaire

Following data synthesis of the included studies and an examination of reflections from the expert advisors, answers to the two research questions (see below) were very broad:

1. What does existing research tell us about self-evaluation as a characteristic of adolescent depression, and an active ingredient in treatment for adolescent depression?
2. To what extent does the existing research reflect the lived experience of self-evaluation and depression, according to experts by experience?

For this reason, findings were grouped under three topic headings: 1) *What does it look like?* 2) *Where does it come from?* And 3) *How can we change it?* Findings from the scoping review are presented below in a narrative form, with an integration of reflections from the expert advisor reflections.

### Topic One – What Does It Look Like?: *“You Forget About All the Good Things, You’d Think, I’m Not Good at Anything” (YP)*

The topic of ‘what does it look like?’ addresses self-evaluation as a *characteristic* of adolescent depression (RQ1), as well as how this aligns with advisor experiences (RQ2).

Thirty studies examined cross-sectional self-evaluation data in depressed samples, consistently reporting a critical view of self, and a reduced positive view. Although there was some evidence of a retained positive view of self, particularly in relation to prosocial traits such as being kind, trustworthy [45]. A number of studies found differences in the types of self-evaluation affected, reflecting the complexity of self-evaluation. For example, Dozois et al. [36] found that depressed adolescents demonstrated worse core beliefs of ‘disconnection’ (e.g. emotional inhibition, social isolation), ‘impaired autonomy’ (e.g. dependence, failure, dependence), and ‘impaired limits’ (e.g. insufficient self-control), but not for ‘exaggerated standards’ (e.g. self-sacrifice, unrelenting standards). Whereas, Koenig [40] found that depressed adolescents had lower self-image on ‘negative image’ (body and self image) and ‘introversion’ (social relationships), but not for ‘unmotivated’ (vocational and educational attitudes) and ‘maladjustment’ (self-reliance).

#### Expert advisory reflections

Consistent with the literature, there was agreement across advisors that both negative and positive self-evaluation are affected during depression. Young people used the terms: “*useless*”, “*unimportant*”, “*all my fault*”, “*not good enough*”, “*doing everything wrong*”, and highlighted an important role for negative evaluations of physical appearance, specifically weight, attractiveness, and acne. All groups also highlighted an overall lack of positive self-evaluation during depression; “*I always see the weaknesses in myself, and I never really see the strengths” (young person)*, but in some cases, prosocial, positive self-evaluations remained, such as being “*kind*” or “*helpful*”, which is consistent with the findings from Orchard et al. [45]

All advisory groups emphasised the complex nature of self-evaluation, and that it varies across and between individuals. They raised how other comorbidities and chronic health issues can influence self-evaluation. Researchers discussed how self-evaluation is affected not only by content of thoughts, but also by their frequency and the value placed on different traits, “*a lot of the young people I work with clinically, the extent to which they self-evaluate and the frequency, is often incredibly elevated relative to people who are not struggling with depression … it seems to be something to do with the frequency of self-evaluation as well as the content of it that becomes problematic*” *(researcher)*. The frequency or value of self-evaluative cognitions were not considered in any identified studies, suggesting a gap in the existing literature.

Young people highlighted the role of others in their self-evaluations, including worrying about how others view them, *“I would worry that I was being boring or not funny, and I’d think that maybe they were just pitying me when they were being friends with me”* (*young person*). They also described a scrutiny of ‘ideals’ and thinking that others are better. It was noted that societal comparisons are often biased by the young person’s own perception; “*because I’ve never really had much input of what people actually think of me, it’s more me just doubting it and being scared that people think negative stuff of me, which backs up the negative things that I think about myself*” (*young person*). Young people also discussed not wanting to bring others down, *“[you] detach yourself from everything else because you don’t want those people to be impacted by the way you feel, and that’s why a lot of people hide it, because they don’t want it rubbing off on anyone else” (young person).*

### Topic Two – Where Does It Come From?: *“I think it’s really hard to view things in isolation because everything, like your sense of yourself and outside events are impacting on you … they are so interlinked and interwoven” (young person)*

The topic of ‘where does it come from?’ addresses how self-evaluation influences depression, and vice versa, as part of self-evaluation as a *characteristic* of adolescent depression (RQ1), as well as how this aligns with advisor experiences (RQ2). Five studies examined the prospective relationship between depression and self-evaluation. These indicated that elevated depression symptoms significantly increase risk of poor self-concept in the future [57, 58], and individuals with a poorer self-image were at increased risk of future depression [59–61], suggesting a possible bidirectional relationship.

#### Expert advisory reflections

The advisory groups discussed the mechanisms by which self-evaluation and depression are causally linked, which was a gap in the literature.

All advisory groups emphasised how self-evaluation is influenced by a wider context, including the social environment; “*self-evaluation doesn’t exist in its own right within that individual, it seems to be shaped by individuals around us*” (*clinician*). Young people described numerous social influences including bullies, social media, stereotypes, parents, and peer comparisons; “*when bullying became a huge part of my life, that sort of just changed the way I viewed myself because I saw it as the truth*” (*young person*). Young people also discussed how gender stereotypes and mental health stigma can cause negative self-evaluation, “*When people talk about depressed people in a condescending way, describing them as “lazy” it feeds back into the cycle of negative self-talk*” (*young person*). Relatedly, they discussed perceived judgment from families; “*I wanted to talk about my feelings but I can’t really do that because my family would be mad*” (*young person*), and “*I’ve heard parents, grandparents, saying ‘you don’t want to be friends with them, they’ve got issues*” (*young person*).

Young people also discussed the influence of cognitive cycles such as over-thinking and a lack of motivation; “*you don’t have any motivation either so then you’re not as productive … Now in society your productivity kind of measures your worth, so if you’re not productive you don’t have any worth in a way*” (*young person*), and behavioural cycles that can exaggerate the problem, such as self-neglect, “*When you’re feeling low, it knocks your mental space into just attacking yourself, you care less about yourself and that’s what you need to do most*” (*young person*).

### Topic Three - How can we change it?: *“I’m pretty sure everyone’s view of themselves could change if they’re getting better and if it doesn’t then I don’t think they’re getting better” (young person)*

The topic of ‘how can we change it?’ reflects the effect of treatment for depression on self-evaluation, directly addressing self-evaluation as an active ingredient in treatment for adolescent depression (RQ1), as well as how this aligns with advisor experiences (RQ2). Eleven studies examined changes in self-evaluation across treatment for young people with depression. Ten of these examined pre- to post- measures of self-evaluation, using varying measurement tools. The remaining article was a case study describing the use of long-term psychodynamic therapy working with self-criticism amongst other difficulties [67].

All psychological intervention studies indicated improvements in self-evaluation for at least one of the interventions examined. However, findings relating to medication were mixed. One study examined medication compared to placebo and did not find an improvement in self-evaluation [68]. Another examined the combined effect of CBT with medication [65], and found that after 12 weeks, the combined treatment group outperformed all other groups on self-concept. Some studies reported between-group differences when comparing effects of different psychological interventions. Fine et al. [64] found a therapeutic support group evidenced greater improvements in self-concept compared to a social skills group post-treatment, but at 9 month follow up the social skills training group had caught up. Rosello et al. [73] found a greater improvement in self-concept for those who received CBT compared to Interpersonal Psychotherapy (IPT). One small, uncontrolled study reported that e-CBT showed improvements pre- to post-treatment, but face to face CBT did not [63].

In relation to domain-specific self-evaluation, two studies examined the effects on subscales: Hintikka et al. [66] found depressed young people showed improvements after treatment on psychological self-image but not familial self-image or all aspects of social self-image. King et al. [62] found that amongst hospitalized young people improvements were only seen in self-perception of social acceptance and global self-worth.

One intervention study reported that although depression severity improved across ages and treatment groups, younger participants showed greater improvements in self-concept [71]. This might suggest it is harder to shift self-evaluations in older participants perhaps due to a stabilising of self-concept. Furthermore, given depression improved in the waitlist control, it is possible that although depression can improve without direct intervention, to change self-concept, intervention is needed, particularly in older participants.

#### Expert advisory reflections

Key reflections that emerged included: the importance of addressing self-evaluation; directly vs indirectly targeting self-evaluation in treatment; and potential intervention approaches. Whilst not all of the reflections directly related to self-evaluation as an ‘active ingredient’, i.e. barriers and facilitators, they do highlight important areas for consideration in future work.

In line with the literature, young people agreed that view of self can change in treatment, describing *“more confidence in self”, “more self-esteem”, “spending more time on self”* and “*acknowledging the positives within themselves”*. Young people explained that self-evaluation should be targeted in treatment and that it is currently not targeted enough. They felt addressing it was important, because it is a “*big part of depress*ion”, and a possible “*underlying factor*”. They also expressed that not addressing self-evaluation could lead to harm itself, and that *“seeing progress in self-evaluation can lead to a more optimistic outlook”* for treatment. However, some young people raised concerns about it needing to be approached with caution.

Researchers and clinicians highlighted that change in self-evaluation could occur via implicit targeting as well as direct intervention, *“[I] … would not talk to them about self-evaluation/esteem explicitly, but prompting questions are important in how they view themselves*” (*clinician*). All groups suggested a number of potential treatment approaches for working with self-evaluation, these included: psycho-education, CBT, mindfulness, compassion-focussed therapy, ACT, value-based approaches, counselling, creative writing and antidepressants. In particular, all groups suggested that relationship focussed interventions e.g. IPT might be helpful. This contradicts findings from Rosello et al. [73], who found better results for CBT. Young people also highlighted that working on their sense of self might feel less pressured in an online format. Interestingly, this supports one study that found better effects for CBT in an online format [63].

Young people discussed the importance of an individualised approach, “*Different techniques help different people so I think letting the person know they can express themselves in any way to help them like writing or drawing*” (*young person*).

#### Barriers and facilitators

All advisory groups talked about barriers to working on self-evaluation in treatment. Researchers and clinicians highlighted that working with ‘self’ takes time and that most current treatments are short-term and self-evaluation is not a priority, “[*there is a] real urgency to offer short term intervention, see people and get people through the door quickly so that we can protect our other services for people who are more unwell*” (*clinician*). Researchers and clinicians discussed cognitive change over time and the ability to access and address thoughts about the self. They considered how “*identity formation*” is fluid during youth, potentially stabilising across adolescence. Young people also felt that a “*deep-rooted*” negative self-evaluation might be difficult to target. This aligns with findings from Rickhi et al. [71] where older participants showed less change in self-concept.

Young people highlighted additional barriers including limitations of using questionnaires to assess self-evaluation, as well as stigma and judgment, particularly from the therapist and family. They also discussed how certain therapist characteristics may make it difficult to discuss self-image, “*I was saying that I thought I was fat one day, and then, because my therapist was quite a large lady, I would always think oh I’m being rude because obviously I’m a lot smaller than her*” (*young person*).

Clinicians and young people talked about facilitators for working on self-evaluation in treatment. Clinicians highlighted “*creating a non-judgemental space that they’re able to talk about stuff, whatever that stuff may be*” (*clinician*), with feelings of safety and trust. Young people also noted some overlapping themes including good therapeutic alliance and trust, “*Talking on a regular basis is the first step for treatment I think. When you talk a lot you develop a strong bond and then you open up more and learn that you won’t get judged and you can talk about whatever you want*.” (*young person*).

## Discussion

This methodologically novel review examined self-evaluation as a characteristic, and active ingredient in treatment, for adolescent depression. Specifically, research questions asked 1) What does existing research tell us about self-evaluation as a characteristic of adolescent depression, and as an active ingredient in treatment for adolescent depression? and 2) To what extent does the existing research reflect the lived experience of self-evaluation and depression, according to experts by experience? A scoping review was conducted and expert advisory groups (researchers, clinicians and young people with lived experience) consulted on their experiences of self-evaluation. The advisory views were integrated with findings from peer-reviewed journal articles, enabling potentially important gaps in the existing literature to be identified. This integrated approach identified three key self-evaluation topics that addressed both research questions simultaneously; ‘What does it look like?’, ‘Where does it come from?’ and ‘How can we change it?’

### Summary of integrated findings

The expert advisors strongly believed that self-evaluation was a key component of depression for young people. Regarding ‘What does it look like?’ advisors agreed with the literature that young people view themselves more negatively and less positively when depressed, however advisors clarified that the view of self is complex and varies for each individual. For ‘Where does it come from?’, the literature examined a prospective relationship, with initial evidence of a bidirectional relationship between depression and self-evaluation. Advisors explored the mechanisms involved in this relationship relating to cognitive development (linking to broader literature on identity development [20, 21]), the social environment, and cognitive and behavioural negative cycles. Minimal literature on mechanisms was identified in our scoping review. Such work often begins with general population samples, where there are some emerging findings (e.g. using mood induction [74]). Future mechanistic research involving young people experiencing elevated depression is a key priority.

There was a consensus from the literature and expert advisors that self-evaluation can improve across treatment. However, research literature was limited, with only 11 identified studies covering a diverse range of interventions and self-evaluation measures.

Self-evaluation was rarely reported as an explicit treatment target, however, this does not necessarily mean that it has not been considered in therapy (e.g. in CBT, cognitive restructuring may be used to improve negative core beliefs about the self). It was clear from our advisors and literature that self-evaluation is covered in some theoretical models and intervention experiences, however, the advisors suggested that more direct emphasis on self-evaluation in treatment may be helpful. They also discussed various barriers and facilitators to working on self-evaluation, such as time, importance of trust, as well as suggestions for possible treatment approaches and techniques that might improve self-evaluation.

### In the context of the wider literature

The findings of this review fit with the original cognitive model of depression which notes a key role for negative thoughts regarding the self [12], with consistent evidence of increased negative and decreased positive self-evaluations. Whilst it remains unclear whether there is a causal relationship between self-evaluation and depression, emerging evidence suggests there might be a bi-directional prospective association. Furthermore, although qualitative research is still substantially lacking in this area (with this review identifying only two qualitative studies), the reflections from the advisory groups supported the importance placed on self-evaluation in other research areas [23].

Whilst literature is yet to provide a strong developmental perspective to the relationship between depression and self-evaluation, e.g. whether the relationship changes over time. There is some evidence to suggest that adolescents at different ages may respond differently to intervention of self-evaluative thoughts, and this may fit with existing knowledge regarding the development and consolidation of a ‘sense of self’ [20].

### Clinical and research implications

Although limited available evidence prevents firm treatment recommendations, our findings suggest that targeting self-evaluation as an active ingredient in treatment is likely to be complex. There appeared to be consensus that the experience and role of self-evaluation is unique and therefore interventions should be individualised. This was reflected in feedback from one young person advisor, who noted that self-evaluation was part of their assessment experience, but that “*There needs to be a focus on allowing the young person to address what they think is the most important issue for themselves*”. Exactly how self-evaluations are targeted for improvement as part of such interventions is unclear, inconsistent and requires further exploration. Further research is needed to understand the nature of self-evaluation and change throughout different types of therapy, to establish whether direct intervention is needed. As recognised by advisors, targeting self-evaluation should be done cautiously and collaboratively with the young person.

This scoping review and the advisory reflections highlight a number of areas for future research, in addition to next steps for intervention. The advisory groups described various experiences that had not been examined in the literature, for example, the importance of the frequency of the self-evaluations, and not just the content. This might reflect an experience of rumination, particularly with regards to self-evaluative thoughts. Rumination is robustly evidenced to be involved in the onset and maintenance of depression [75, 76], but is often considered to reflect broader negative thoughts. Adolescence is a time of critical development of self-concept with the theorised development of abstract self-portraits, internalised standards (i.e. self-generated expectations) and the integration of multiple selves into a unified self-concept [77]. As such, it may be that adolescents are particularly vulnerable to self-evaluative rumination. Future work would benefit from further examination of the frequency of negative self-evaluative thoughts, but it would also be interesting to examine whether this is unique to adolescents, or perhaps more prevalent amongst adolescents compared to adults.

### Strengths and limitations

This is the first review to examine the broader literature under the umbrella term ‘self-evaluation’ in adolescent depression. The novel review strategy has some important strengths. Firstly, the inclusion of expert advisor input allows literature to be embedded in the real world, identifying richer and more diverse details about self-evaluation than those only described in published research studies. A traditional scoping review would rely on the author’s opinion regarding what important questions remain to be answered in this area. The methodology adopted here meant that the review could draw on a much wider range of expertise to make these judgements. Finally, the wide range of ‘self’ terms included in the systematic search allowed us to identify a diverse range of studies in this field. Whilst this presents challenges to the examination of literature in the field, e.g. the use of systematic approaches, initially pulling together these studies into one review will provide a useful first step for other reviews to follow. There are however some limitations to note. Studies in this review were required to include participants with a depression diagnosis or elevated symptom scores, so some studies with correlational and experimental designs were not included. Scoping reviews do not typically include a quality assessment of studies [78] or consideration of publication bias. Self-evaluation research is an emerging field and we hope this review will be a catalyst for many more studies on this important topic. However, due to the limited scope of the field, it was difficult to examine the role of self-evaluation as an ‘active ingredient’ in treatment as there were limited studies addressing this topic. This meant that the first research question was only partially answered. More primary research is needed examining whether improving self-evaluation improves outcomes for depression. Furthermore, future systematic reviews in the field will be needed and should include study quality and publication bias to help evaluate the evidence.

## Conclusions

This novel scoping review identifies the importance and complexity of self-evaluation in adolescent depression, revealed by existing research and the views of expert advisors. Young people with depression experience impaired self-evaluation that can change over time, is complex in nature, and can improve with treatment. However, much more work is needed to understand the casual relationship between self-evaluation and depression in adolescence, improve and standardise self-evaluation measurement, and investigate the role of self-evaluation in treatment. We believe that to fully understand and improve outcomes for depressed adolescents, research needs to involve young people with lived experience as active stakeholders. We hope that this approach will continue to be embedded in future work.

## Supplementary Information

**Additional file 1.**

## Data Availability

All data generated or analysed during this study are included in this published article [and its supplementary information files].
