# Supplementary material for: Self-evaluation as an active ingredient in the experience and treatment of adolescent depression; an integrated scoping review with expert advisory input
Source: BMC Psychiatry. 2021 Dec 3;21:603. doi: 10.1186/s12888-021-03585-5 (PMC8641228; doi:10.1186/s12888-021-03585-5)
Supplement: Supplementary file 1 — Additional file 1. [file 12888_2021_3585_MOESM1_ESM.docx]

Prospero – pre-registration

Wellcome study – self-evaluation in adolescent depression

| # | Section | Mandatory? | Answer/details required |
| --- | --- | --- | --- |
| 1 | Review Title | Yes | Self-evaluation as a characteristic and target of intervention in adolescent depression; a systematic scoping review |
| 2 | Original language title | No |  |
| 3 | Anticipated or actual start date | yes | Date in the format eg 01 July 2020 |
| 4 | Anticipated completion date | yes | Date in the format eg 30 September 2020 |
| 5 | Stage of review at time of this submission | yes |  |
| 6 | Named contact | yes | Dr Faith Orchard |
| 7 | Named contact email | Yes | [f.orchard@reading.ac.uk](mailto:f.orchard@reading.ac.uk) |
| 8 | Named contact address | No | School of Psychology and Clinical Language Sciences, University of Reading, Reading, RG6 6AL |
| 9 | Named contact phone number | No |  |
| 10 | Organisational affiliation of the review | Yes | School of Psychology and Clinical Language Sciences, University of Reading |
| 11 | Review team members and their organisational affiliations | Yes | Dr Faith Orchard, School of Psychology and Clinical Language Sciences, University of Reading  Dr Brioney Gee, Norfolk and Suffolk NHS Foundation Trust  Dr Tim Clarke, Norfolk and Suffolk NHS Foundation Trust  Dr Laura Pass, University of East Anglia.  Juliette Westbrook, School of Psychology and Clinical Language Sciences, University of Reading  Sophie Allan, University of East Anglia |
| 12 | Funding sources/sponsors | Yes | This work was funded by a Wellcome Trust Mental Health Priority Area 'Active Ingredients' commission awarded to University of Reading. |
| 13 | Conflicts of interest | Yes | There are no conflicts of interest |
| 14 | Collaborators |  | None. |
| 15 | Review question | Yes | 1) what is known about self-evaluation as a *characteristic* of adolescent depression?  2) what is known about self-evaluation as a *target of intervention* for adolescent depression? |
| 16 | Searches | Yes | Studies will be identified through electronic database searching using a pre-piloted search string tailored to each database combining synonyms for self-evaluation, depression and adolescents using Boolean operators. Search terms were finalised following circulation of a brief online survey to researchers working in relevant fields, inviting their input on terminology to include. An example search string developed for Web of Science is provided below:  (self-evaluat* OR self-concept OR self-worth OR self-aware* OR self-inhibiting OR "view of self" OR self-assessment* OR "positive evaluation" OR "negative evaluation" OR "positive self" OR "negative self" OR self-reflect* OR self-description OR cognitive-evaluation OR "self-referential processing" OR self-criticism OR self-perception OR self-cognition OR "cognitions about the self" OR self-schema* OR self-image OR "sense of self" OR self-identity OR self-representation OR self-belief* OR self-efficacy OR self-hat* OR self-appraisal)   AND  (depress* OR   MDD  OR  "low  mood")   AND  (adolescen*OR  teen*  OR  youth*  OR  young OR  student*  OR  child*  OR  pupil*  OR  juvenile*  OR  "emerging  adult")  We will not limit the search by year of publication or language (to enable us to check for an English language version of any potentially relevant non-English publications identified). We intend to search the following databases:   1. PsychInfo 2. EMBASE 3. Web of Science 4. Medline 5. The Cochrane Library   Reference lists of eligible papers will be hand searched to ensure that no relevant papers have been missed. References of any relevant reviews identified during the screening process will also be searched for potentially relevant primary research. |
| 17 | URL to search strategy | No |  |
| 18 | Condition or domain being studied | Yes | Self-evaluation in adolescent depression.  We are conceptualising self-evaluation as the perceptions and beliefs which a young person holds about themselves, specifically the emotionally-valenced qualities, characteristics and traits (both positive and negative), and the young person’s judgement of the value of these attributes. |
| 19 | Participants/population | Yes | Adolescents (aged 11 to 24) with a diagnosis of, or exceed an elevated clinical threshold for, depression/low mood |
| 20 | Intervention(s), exposure(s) | Yes | This review will not be examining specific interventions, but will include studies that examine ‘self-evaluation’ as part of intervention studies. |
| 21 | Comparator(s)/control | Yes | Not applicable |
| 22 | Types of study to be included | Yes | Both quantitative and qualitative studies of any design will be eligible for inclusion provided they meet the following inclusion criteria and do not meet any of the exclusion criteria below.  Inclusion:   - Paper published in a peer review journal - Paper must report the findings of primary research - Participants aged between 11-24 years (all participants are reported to be aged 11-24 or, where only mean and standard deviation (SD) are reported, the mean age +/-SD falls within this range) - Participants (or at least one group of participants for which data is reported separately) have a primary diagnosis of depression or meet a threshold for elevated depression symptoms prespecified by the study authors. - Studies that report on self-evaluation as a characteristic, or focus of treatment, in adolescent depression   Exclusion:   - Papers that measure self-esteem or self-efficacy in relation to a specific skill/domain only, and do not contain any other measure of self-evaluation - Participants are included in the study on the basis of a physical health problem, social grouping (e,g, refugee status, homelessness), or a mental health condition other than depression or anxiety (due to high co-morbidity with depression). - Abstract (including conference abstracts and poster presentations) or protocol only - Grey literature - Systematic reviews and meta analyses - Full text not available in English. |
| 23 | Context | No |  |
| 24 | Main Outcome(s) | Yes | The review will aim to identify what is known about ‘self-evaluation’ as a characteristic of adolescent depression, and to identify whether ‘self-evaluation’ changes after intervention for depression in adolescents. |
| 25 | Additional outcome(s) | Yes | To establish to what extent does the existing research reflects the lived experience of self-evaluation and depression, according to experts by experience |
| 26 | Data extraction (selection and coding) | Yes | The results of the data base searches will be imported into Covidence. An initial screening of the first 5% of the titles and abstracts will be blindly carried out by two researchers in accordance with a pre-specified inclusion criteria. We will review concordance and clarify any ambiguity in the eligibility before proceeding. Disagreements will be checked by a third reviewer.  Full texts will be reviewed for all papers that are potentially relevant, based on title and abstract screen  Data extraction will be carried out by at least two members of the research team independently.  The number of studies obtained from the search, and included and excluded at each stage of the review process will be recorded using a PRISMA flow diagram.  The following data will be extracted: participant demographics and characteristics, measure of depression and mean depression score, measure of ‘self-evaluation’ and mean scores or qualitative discussions and themes (as applicable), intervention type (if applicable), change in ‘self-evaluation’ (if applicable). Two reviewers will extract and check the data. Data will be recorded in Covidence. |
| 27 | Risk of bias (quality) assessment | Yes | The primary purpose of this commissioned scoping review is to identify the extent of the existent literature on self-evaluation as a characteristic, or focus of treatment, in adolescent depression, rather than the establish quality and reliability of a specific type of study or outcome. As such, risk of bias is not directly relevant to the focus of the review. Furthermore, rigour and quality is being assessed by examining how the existing literature fits with the views and ideas of stakeholders (see data analysis). |
| 28 | Strategy for data analysis | Yes | Scoping reviews aim to map key concepts, types of evidence and gaps in research related to a defined research topic by systematically searching, selecting and synthesising existing literature. To achieve these aims, our approach to data synthesis will combine descriptive quantitative summary of the extent of the literature with qualitative narrative summary of study findings. In line with published recommendations for scoping reviews (Arksey et al., 2005; Levac et al., 20120) and the requirements of the funder, the insights of stakeholders, including young people with lived-experience of depression, will be integrated with the narrative summary. |
| 29 | Analysis of subgroups or subsets | Yes | There are no planned subgroup analyses. |
| 30 | Type and method of review | yes | Scoping review |
| 31 | Language | no | English (default) |
| 32 | Country | yes | England |
| 33 | Other registration details | no |  |
| 34 | Reference and/or URL for published protocol | No |  |
| 35 | Dissemination plans | No | The findings will be shared in a report/publication, and in a number of accessible public facing documents, as part of the Wellcome Trust project. |
| 36 | Keywords | No | Adolescence; self-evaluation; depression |
| 37 | Details of any existing review of the same topic by the same authors | no |  |
| 38 | Current review status | yes | Complete |
| 39 | Any additional information | no |  |
| 40 | Details of final report/publication(s) or preprints if available | NA |  |
